# Convergence Analysis and Numerical Study of a Fixed-Point Iterative Method for Solving Systems of Nonlinear Equations

**DOI:** 10.1155/2014/789459

**Published:** 2014-03-24

**Authors:** Na Huang, Changfeng Ma

**Affiliations:** School of Mathematics and Computer Science, Fujian Normal University, Fuzhou 350007, China

## Abstract

We present a fixed-point iterative method for solving systems of nonlinear equations. The convergence theorem of the proposed method is proved under suitable conditions. In addition, some numerical results are also reported in the paper, which confirm the good theoretical properties of our approach.

## 1. Introduction

One of the basic problems in mathematics is how to solve nonlinear equations

(1) $$\begin{array}{c} f\left(x\right)=0. \end{array}$$

In order to solve these equations, we can use iterative methods such as Newton's method and its variants. Recently, there has been some progress on iterative methods with higher order of convergence using decomposition techniques; see [1–15] and the reference therein.

The zeros of a nonlinear equation cannot in general be expressed in closed form; thus we have to use approximate methods. Nowadays, we often use iterative methods to get the approximate solution of the system (1); the best known method is the classical Newton's method. Recently, there has been some progress on solving the system (1), which allows us to get the iterative formula by using essentially Taylor's polynomial (see [16, 17]), quadrature formulas (see [7, 9–12]), homotopy perturbation method (see [8]), and so on.

In this paper, we will present a new fixed point iterative method for solving the system (1) and prove that the method is cubic convergent under suitable conditions.

This paper is organized as follows. In Section 2, we introduce new iterative methods to solve (1). In Section 3 we extend these methods to solve systems of nonlinear equations, and we also prove convergence of the proposed method. Some numerical results are reported in Section 4, while the paper is concluded in the last section.

## 2. Iterative Methods and Convergence Analysis

We now consider the following nonlinear equation:

(2) $$\begin{array}{c} f\left(x\right)=0,\;x\in \left[a,b\right]. \end{array}$$

Assume that α* is a simple root of (2); that is, *f*(α*) = 0. For *α*, *α*_*k*_ ∈ [*a*, *b*], using Taylor's formula, we have

(3) f(α)=f(αk)+f′(αk)(α−αk)+12!f′′(αk)(α−αk)2+13!f′′′(αk)(α−αk)3+⋯+1(r−1)!f(r−1)(αk)(α−αk)r−1+∫01 (1−t)r−1(r−1)!f(r)(αk+t(α−αk))(α−αk)rdt.

Taking *r* = 1 in the above equality, we get

(4) $$\begin{array}{c} f\left(\alpha \right)=f\left(\alpha_{k}\right)+\int_{0}^{1}f^{\prime }\left(\alpha_{k}+t\left(\alpha -\alpha_{k}\right)\right)\left(\alpha -\alpha_{k}\right)\text{d}t. \end{array}$$

If the value of *f*′(*α*_*k*_ + *t*(*α* − *α*_*k*_)) in the interval [0,1] is replaced with its value in *t* = 0, that is, with *f*′(*α*_*k*_), then we have

(5) $$\begin{array}{c} \int_{0}^{1}f^{\prime }\left(\alpha_{k}+t\left(\alpha -\alpha_{k}\right)\right)\left(\alpha -\alpha_{k}\right)\text{d}t≅f^{\prime }\left(\alpha_{k}\right)\left(\alpha -\alpha_{k}\right). \end{array}$$

By using (5) in (4), we have

(6) $$\begin{array}{c} f\left(\alpha \right)≅f\left(\alpha_{k}\right)+f^{\prime }\left(\alpha_{k}\right)\left(\alpha -\alpha_{k}\right). \end{array}$$

We can get an iterative method from (6) to solve the system (2); it is the famous Newton's formula

(7) $$\begin{array}{c} \alpha_{k+1}=\alpha_{k}-\frac{f\left(\alpha_{k}\right)}{f^{\prime }\left(\alpha_{k}\right)}. \end{array}$$

The formula (7) has already been proved to be quadratically convergent. Now we begin to deduce a higher order iterative method. In fact, if we estimate *f*′(*α*_*k*_ + *t*(*α* − *α*_*k*_)) in the interval [0,1] by its value in *t* = 1, that is, by *f*′(*α*), then we have

(8) ∫01f′(αk+t(α−αk))(α−αk)dt  =f′(α)(α−αk)+R[f(α)].

By using (8) in (4), and cutting off the error *R*[*f*(*α*)] (still use “=”), we have

(9) $$\begin{array}{c} f\left(\alpha \right)=f\left(\alpha_{k}\right)+f^{\prime }\left(\alpha \right)\left(\alpha -\alpha_{k}\right). \end{array}$$

Let *α*_*k*+1_ be the solution of (9); we can obtain a new iterative method

(10) $$\begin{array}{c} \alpha_{k+1}=\alpha_{k}-\frac{f\left(\alpha_{k}\right)-f\left(\alpha_{k+1}\right)}{f^{\prime }\left(\alpha_{k+1}\right)}. \end{array}$$

Since the iterative method (10) is implicit-type method, we use classical Newton's formula (7) as predictor and then use the above scheme (10) as corrector; in this way, we can get a workable iterative method.

**Algorithm 1**

*Step 0 (initialization)*. Choose the initial value *α*_0_ ∈ *N*(α*, *δ*), where α* is certain real zero of nonlinear mapping *f*(*α*) and *δ* > 0 is a sufficiently small constant. Take the stopping criteria *ϵ*_1_, *ϵ*_2_ > 0. Set *k* : = 0.

*Step 1 (the predictor step)*. Compute the predictor

(11) $$\begin{array}{c} \beta_{k}=\alpha_{k}-\frac{f\left(\alpha_{k}\right)}{f^{\prime }\left(\alpha_{k}\right)}. \end{array}$$

*Step 2 (the corrector step).* Computing the corrector

(12) $$\begin{array}{c} \alpha_{k+1}=\alpha_{k}-\frac{f\left(\alpha_{k}\right)-f\left(\beta_{k}\right)}{f^{\prime }\left(\beta_{k}\right)}. \end{array}$$

*Step 3*. If |*f*(*α*_*k*+1_)|≤*ϵ*_1_ or |*α*_*k*+1_ − *α*_*k*_ | ≤*ϵ*_2_ then stop; otherwise, set *α*_*k*_ : = *α*_*k*+1_, *k* : = *k* + 1; go to Step 1.

In this section, we consider the convergence and convergent rate of Algorithm 1. We obtain a convergence theorem as follows.

**Theorem 2**

Let α* ∈ *I* be a simple zero of sufficiently differentiable function *f* : [*a*, *b*]⊆*R* → *R*. If *α*_0_ is sufficiently close to α*, then the two-step iterative method defined by (11)-(12) has three-order convergence and satisfies the following error equation:

(13) $$\begin{array}{c} e_{k+1}=c_{1}e_{k}^{3}+O\left(e_{k}^{4}\right), \end{array}$$

where *e*_*k*_ = *α*_*k*_ − α* and *c*_1_ = *f*′′′(α*)/6*f*′(α*).

**Proof**

By (12) we get

(14) $$\begin{array}{c} e_{k+1}=e_{k}-\frac{f\left(\beta_{k}\right)-f\left(\alpha_{k}\right)}{f^{\prime }\left(\beta_{k}\right)}. \end{array}$$

Multiplying the above equation by *f*′(*β*_*k*_), we can get

(15) $$\begin{array}{c} f^{\prime }\left(\beta_{k}\right)e_{k+1}=f^{\prime }\left(\beta_{k}\right)e_{k}+\left[f\left(\beta_{k}\right)-f\left(\alpha_{k}\right)\right]. \end{array}$$

Let *α* = α* in (3) and *f*(α*) = 0; we have

(16) 0=f(αk)+f′(αk)(α∗−αk)+12!f′′(αk)(α∗−αk)2+13!f′′′(αk)(α∗−αk)3+O((α∗−αk)4);

thus,

(17) $$\begin{array}{c} f\left(\alpha_{k}\right)=f^{\prime }\left(\alpha_{k}\right)e_{k}-\frac{1}{2}f^{\prime \prime }\left(\alpha_{k}\right)e_{k}^{2}+\frac{1}{6}f^{\prime \prime \prime }\left(\alpha_{k}\right)e_{k}^{3}+O\left(e_{k}^{4}\right). \end{array}$$

Dividing both sides of the above equation by *f*′(*α*_*k*_), we can get

(18) f(αk)f′(αk)=ek−12f′′(αk)f′(αk)ek2+16f′′′(αk)f′(αk)ek3+O(ek4)=ek−12f′′(αk)f′(αk)ek2+O(ek3).

Furthermore, let *α* = *β*_*k*_ in (3); we have

(19) f(βk)=f(αk)+f′(αk)(βk−αk)+12!f′′(αk)(βk−αk)2+13!f′′′(αk)(βk−αk)3+O((βk−αk)4).

From (11) we get

(20) $$\begin{array}{c} \beta_{k}-\alpha_{k}=-\frac{f\left(\alpha_{k}\right)}{f^{\prime }\left(\alpha_{k}\right)}. \end{array}$$

It follows from the above equation that

(21) f(βk)=f(αk)−f′(αk)[f(αk)f′(αk)]+12f′′(αk)[f(αk)f′(αk)]2−16f′′′(αk)[f(αk)f′(αk)]3+O([f(αk)f′(αk)]4)=12f′′(αk)[f(αk)f′(αk)]2−16f′′′(αk)[f(αk)f′(αk)]3+O([f(αk)f′(αk)]4).

By applying (18) we have

(22) f(βk)=12f′′(αk)[ek−12f′′(αk)f′(αk)ek2+O(ek3)]2−16f′′′(αk)[ek−12f′′(αk)f′(αk)ek2+O(ek3)]3+O(ek4).

After some manipulations we obtain

(23) f(βk)=12f′′(αk)ek2−12[f′′(αk)]2f′(αk)ek3−16f′′′(αk)ek3+O(ek4).

Now, applying Taylor's expansion for *f*′(*β*_*k*_) at the point *α*_*k*_, we have

(24) f′(βk)=f′(αk)+f′′(αk)(βk−αk)+12!f′′′(αk)(βk−αk)2+O((βk−αk)3).

From (11), (18), and (24), we obtain

(25) f′(βk)=f′(αk)−f′′(αk)[ek−12f′′(αk)f′(αk)ek2+O(ek3)]+12f′′′(αk)[ek−12f′′(αk)f′(αk)ek2+O(ek3)]2+O(ek3).

And after some manipulations we can get

(26) f′(βk)=f′(αk)−f′′(αk)ek+12[f′′(αk)]2f′(αk)ek2+12f′′′(αk)ek2+O(ek3).

By substituting (17), (23), and (26) into (15) we have

(27) f′(βk)ek+1 =f′(βk)ek+[f(βk)−f(αk)] ={f′(αk)−f′′(αk)ek+12[f′′(αk)]2f′(αk)ek2   +12f′′′(αk)ek2+O(ek3)}ek  +{12f′′(αk)ek2−12[f′′(αk)]2f′(αk)ek3    −16f′′′(αk)ek3+O(ek4)}  −{f′(αk)ek−12f′′(αk)ek2    +16f′′′(αk)ek3+O(ek4)} =16f′′′(αk)ek3+O(ek4).

Note that

(28) $$\begin{array}{c} f^{\prime }\left(\beta_{k}\right)=f^{\prime }\left(\alpha^{*}\right)+O\left(e_{k}\right),\\ f^{\prime \prime \prime }\left(\alpha_{k}\right)=f^{\prime \prime \prime }\left(\alpha^{*}\right)+O\left(e_{k}\right). \end{array}$$

Then, it follows from (27) that

(29) ek+1=16f′′′(α∗)+O(ek)f′(α∗)+O(ek)ek3+O(ek4)=16[f′′′(α∗)f′(α∗)+O(ek)]ek3+O(ek4)=16f′′′(α∗)f′(α∗)ek3+O(ek4).

This proves the conclusion of the theorem and the proof is completed.

## 3. The* n*-Dimensional Case

In this section, we consider the *n*-dimensional case of the method, and we also study these iterative methods' order of convergence. Consider the system of nonlinear equations

(30) $$\begin{array}{c} f_{1}\left(x_{1},x_{2},\ldots ,x_{n}\right)=0,\\ f_{2}\left(x_{1},x_{2},\ldots ,x_{n}\right)=0,\\ \vdots \\ f_{n}\left(x_{1},x_{2},\ldots ,x_{n}\right)=0, \end{array}$$

where each function *f*_*i*_(*x*_1_, *x*_2_,…, *x*_*n*_), *i* = 1,2,…, *n*, maps a vector *x* = (*x*_1_, *x*_2_,…, *x*_*n*_)^*T*^ of the *n*-dimensional space *R* ^*n*^ into the real line *R*. The system (30) of *n* nonlinear equations in *n* unknowns can also be represented by defining a function *F* mapping *R* ^*n*^ into *R* ^*n*^ as *F*(*x*) = (*f*_1_(*x*), *f*_2_(*x*),…, *f*_*n*_(*x*))^*T*^. Thus, the system (30) can be written in the form

(31) $$\begin{array}{c} F\left(x\right)=0. \end{array}$$

Let *F* : *D*⊆*R* ^*n*^ → *R* ^*n*^ be a sufficiently differentiable function on a convex set *D*⊆*R* ^*n*^ and let x* be a real zero of the nonlinear mapping *F*(*x*); that is, *F*(x*) = 0. For any *x*, *x*_*k*_ ∈ *D*, we may write Taylor's expansion for *F* as follows (see [17]):

(32) F(x)=F(xk)+F′(xk)(x−xk)+12!F′′(xk)(x−xk)2+13!F′′′(xk)(x−xk)3+⋯+1(r−1)!F(r−1)×(xk)(x−xk)r−1+∫01 (1−t)r−1(r−1)!F(r)(xk+t(x−xk))(x−xk)rdt;

for *r* = 1, we have

(33) $$\begin{array}{c} F\left(x\right)=F\left(x_{k}\right)+\int_{0}^{1}F^{\prime }\left(x_{k}+t\left(x-x_{k}\right)\right)\left(x-x_{k}\right)\text{d}t. \end{array}$$

If we estimate *F*′(*x*_*k*_ + *t*(*x* − *x*_*k*_)) in the interval [0,1] by its value in *t* = 0, that is, by *F*′(*x*_*k*_), then we have

(34) $$\begin{array}{c} \int_{0}^{1}F^{\prime }\left(x_{k}+t\left(x-x_{k}\right)\right)\left(x-x_{k}\right)\text{d}t≅F^{\prime }\left(x_{k}\right)\left(x-x_{k}\right). \end{array}$$

By using (34) in (33), we have

(35) $$\begin{array}{c} F\left(x\right)≅F\left(x_{k}\right)+F^{\prime }\left(x_{k}\right)\left(x-x_{k}\right). \end{array}$$

We can get an iterative method from (35) to solve the system (31); it is known as Newton's method

(36) $$\begin{array}{c} x_{k+1}=x_{k}-F^{\prime }{\left(x_{k}\right)}^{-1}F\left(x_{k}\right). \end{array}$$

The method (36) has already been proved that it has quadratic convergence. Now we begin to deduce a higher order iterative method. If we estimate *F*′(*x*_*k*_ + *t*(*x* − *x*_*k*_)) in the interval [0,1] by its value in *t* = 1, that is, by *F*′(*x*), then we have

(37) ∫01 ‍F′(xk+t(x−xk))(x−xk)dt  =F′(x)(x−xk)+R[F(x)].

By using (37) in (33), and cutting off the error *R*[*F*(*x*)] (still use “=”), we have

(38) $$\begin{array}{c} F\left(x\right)=F\left(x_{k}\right)+F^{\prime }\left(x\right)\left(x-x_{k}\right). \end{array}$$

Let *x*_*k*+1_ be the solution of (38); we can obtain a new iterative method

(39) $$\begin{array}{c} x_{k+1}=x_{k}-F^{\prime }{\left(x_{k+1}\right)}^{-1}\left[F\left(x_{k}\right)-F\left(x_{k+1}\right)\right]. \end{array}$$

On the other hand, from the easy Newton's method (see [4]), we obtain

(40) xk+1=xk−F′(xk)−1[F(xk)+F(xk+1)].

Now we consider the convex combination of (39) and (40). Let *α* ≥ 0, *β* ≥ 0, and *α* + *β* = 1; then we can deduce from (39) and (40) that the following iterative formula holds:

(41) xk+1=xk−[αF′(xk+1)−1+βF′(xk)−1]×[F(xk)+F(xk+1)]+2αF′(xk+1)−1F(xk+1).

Since the iterative method (41) is implicit-type method, we use Newton's method as predictor and then use the new method (41) as corrector; in this way, we can get a workable iterative method.

**Algorithm 3**

*Step 0 (initialization)*. Choose the initial value *x*_0_ ∈ *N*(x*, *δ*), *α* ∈ [0,1] and *β* = 1 − *α*, where x* is certain real zero of nonlinear mapping *F*(*x*) and *δ* > 0 is a sufficiently small constant. Take the stopping criterions *ϵ*_1_, *ϵ*_2_ > 0. Set *k* : = 0.

*Step 1 (the predictor step)*. Compute the predictor

(42) $$\begin{array}{c} y_{k}=x_{k}-F^{\prime }{\left(x_{k}\right)}^{-1}F\left(x_{k}\right). \end{array}$$

*Step 2 (the corrector step)*. Computing the corrector

(43) xk+1=xk−[αF′(βk)−1+βF′(xk)−1]×[F(βk)+F(xk)]+2αF′(βk)−1F(βk).

*Step 3.* If ||*F*(*x*_*k*+1_)|| ≤ *ϵ*_1_ or ||*x*_*k*+1_ − *x*_*k*_|| ≤ *ϵ*_2_ then stop; otherwise, set *k* : = *k* + 1; go to Step 1.

**Remark 4**

If we take *β* = 0, *α* = 1, our algorithms (42) and (43) can be written in the following form:

(44) $$\begin{array}{c} y_{k}=x_{k}-F^{\prime }{\left(x_{k}\right)}^{-1}F\left(x_{k}\right),\\ x_{k+1}=x_{k}-{\left[F^{\prime }\left(y_{k}\right)\right]}^{-1}\left[F\left(x_{k}\right)-F\left(y_{k}\right)\right]. \end{array}$$

Notice that, at each iteration, the number of functional evaluations is 2*n* ^2^ + 2*n*.

In this section, we consider the convergence and convergent rate of Algorithm 1. We obtain a convergence theorem as follows.

**Theorem 5**

Let *F* : *D*⊆*R* ^*n*^ → *R* ^*n*^ be a sufficiently differentiable function on a convex set *D*⊆*R* ^*n*^ containing a root x* of the nonlinear system (31). The iterative method (42)-(43) has cubic convergence and satisfies the error equation

(45) ek+1=F′(x∗)−1   ×[16αF′′′(x∗)+12βF′′(x∗)F′(x∗)−1     ×F′′(x∗)]ek3+O(||ek4||).

**Proof**

Defining *e*_*k*_ = *x*_*k*_ − x*, from (42) and (43), we have

(46) ek+1=ek−[αF′(yk)−1+βF′(xk)−1]×[F(yk)+F(xk)]+2αF′(yk)−1F(yk).

Premultiplying the above equation by *F*′(*y*_*k*_), we can get

(47) F′(yk)ek+1=F′(yk)ek−[αI+βF′(yk)F′(xk)−1]×[F(yk)+F(xk)]+2αF(yk).

Let *x* = x* in (32) and *F*(x*) = 0; we have

(48) 0=F(xk)+F′(xk)(x∗−xk)+12!F′′(xk)(x∗−xk)2+13!F′′′(xk)(x∗−xk)3+O(||(x∗−xk)4||);

thus,

(49) F(xk)=F′(xk)ek−12F′′(xk)ek2+16F′′′(xk)ek3+O(||ek4||).

Multiplying *F*′(*x*_*k*_)^−1^ to the two sides of the above equation, we obtain

(50) F′(xk)−1F(xk)=ek−12F′(xk)−1F′′(xk)ek2+16F′(xk)−1F′′′(xk)ek3+O(||ek4||)=ek−12F′(xk)−1F′′(xk)ek2+O(||ek3||).

On the other hand, let *x* = *y*_*k*_ in (32); we have

(51) F(yk)=F(xk)+F′(xk)(yk−xk)+12!F′′(xk)(yk−xk)2+13!F′′′(xk)(yk−xk)3+O(||(yk−xk)4||).

From (42) we get *y*_*k*_ − *x*_*k*_ = −*F*′(*x*_*k*_)^−1^ *F*(*x*_*k*_). It follows from the above equation that

(52) F(yk)=F(xk)−F′(xk)[F′(xk)−1F(xk)]+12F′′(xk)[F′(xk)−1F(xk)]2−16F′′′(xk)[F′(xk)−1F(xk)]3+O(||F′(xk)−1F(xk)||4)=12F′′(xk)[F′(xk)−1F(xk)]2−16F′′′(xk)[F′(xk)−1F(xk)]3+O(||[F′(xk)−1F(xk)]4||).

By applying (50) we have

(53) F(yk)=12F′′(xk)×[ek−12F′(xk)−1F′′(xk)ek2+O(||ek3||)]2−16F′′′(xk)×[ek−12F′(xk)−1F′′(xk)ek2+O(||ek3||)]3+O(||ek4||).

After some manipulations we obtain

(54) F(yk)=12F′′(xk)ek2−12F′′(xk)F′(xk)−1F′′(xk)ek3−16F′′′(xk)ek3+O(||ek4||).

Now, applying Taylor's formula for *F*′(*y*_*k*_) at the point *x*_*k*_, we have

(55) F′(yk)=F′(xk)+F′′(xk)(yk−xk)+12!F′′′(xk)(yk−xk)2+O(||(yk−xk)3||).

From (42), (50), and (55), we obtain

(56) F′(yk)=F′(xk)−F′′(xk)×[ek−12F′(xk)−1F′′(xk)ek2+O(||ek3||)]+12F′′′(xk)×[ek−12F′(xk)−1F′′(xk)ek2+O(||ek3||)]2+O(||ek3||).

And after some manipulations we can get

(57) F′(yk)=F′(xk)−F′′(xk)ek+12F′′(xk)F′(xk)−1F′′(xk)ek2+12F′′′(xk)ek2+O(||ek3||).

Therefore, we have

(58) αI+βF′(yk)F′(xk)−1  =I−β[F′′(xk)ek−12F′′(xk)F′(xk)−1      ×F′′(xk)ek2−12F′′′(xk)ek2]F′(xk)−1   +O(||ek3||).

From (49) and (54), we get

(59) F(xk)+F(yk)=F′(xk)ek−12F′′(xk)×F′(xk)−1F′′(xk)ek3+O(||ek4||).

By substituting (57), (58), (59), and (54) into (47) we have

(60) F′(yk)ek+1=F′(yk)ek−[αI+βF′(yk)F′(xk)−1]×[F(yk)+F(xk)]+2αF(yk)=F′(xk)ek−F′′(xk)ek2+12F′′(xk)F′(xk)−1F′′(xk)ek3+12F′′′(xk)ek3+O(||ek4||)−{I−β[F′′(xk)ek−12F′′(xk)     ×F′(xk)−1F′′(xk)ek2     −12F′′′(xk)ek2]  ×F′(xk)−1+O(||ek3||)}×[F′(xk)ek−12F′′(xk)F′(xk)−1  × F′′(xk)ek3+O(||ek4||)]+2α[12F′′(xk)ek2−12F′′(xk)   ×F′(xk)−1F′′(xk)ek3   −16F′′′(xk)ek3+O(||ek4||)]=[16αF′′′(xk)+12βF′′(xk) × F′(xk)−1F′′(xk)]ek3+O(||ek4||);

that is,

(61) ek+1=F′(yk)−1×[16αF′′′(xk)+12βF′′(xk)F′(xk)−1F′′(xk)]×ek3+O(||ek4||).

Notice that

(62) F′(yk)=F′(x∗)+O(ek)F(k)(xk)=F(k)(x∗)+O(ek),k=1,2,3.

Equation (61) can be written as follows:

(63) ek+1=F′(x∗)−1×[16αF′′′(x∗)+12βF′′(x∗)F′(x∗)−1F′′(x∗)]×ek3+O(||ek4||),

which proves the conclusion of our theorem. The proof is completed.

## 4. Numerical Examples

In this section we present some examples to illustrate the efficiency and the performance of the newly developed method (42)-(43) (present study HM). This new method was compared with Newton's method (NM), the method of Aslam Noor and Waseem [12] (NR1), the method of Cordero et al. [13] (NAd1), the method of Darvishi and Barati [4] (DV), and the method of Cordero and Torregrosa [10] (CT) in the number of iterations, CPU time, error, and convergence order. All computations were done using the PC with Pentium(R) Dual-Core CPU T4400 @2.20 GHz. All the programming is implemented in MATLAB 7.9. The convergence order *p* is computed approximately by the following formula:

(64) $$\begin{array}{c} p≅\{\begin{array}{cc} \frac{\ln\left({||x_{k+1}-x_{k}||}_{\infty }/{||x_{k}-x_{k-1}||}_{\infty }\right)}{\ln\left({||x_{k}-x_{k-1}||}_{\infty }/{||x_{k-1}-x_{k-2}||}_{\infty }\right)}, & \text{if}\;\;k\geq 3;\\ +\infty , & \text{otherwise.} \end{array} \end{array}$$

As the iterative formula (43) contains parameters *α* and *β*, we make the numerical examples based on *α* = 1 and *β* = 0.

**Example 1**

The test function is as follows (see [10]):

(65) $$\begin{array}{c} F\left(x\right)={\left(x-1\right)}^{6}-1. \end{array}$$

This problem has a solution x* = 2. We test this problem by using *x*_0_ = 4 as a starting point. The test results are listed in Table 1.

**Example 2**

The test function is as follows (see [9, 10, 13, 14]):

(66) $$\begin{array}{c} F\left(x_{1},x_{2}\right)=\left(\begin{array}{c} x_{1}+e^{x_{2}}-\cos\;x_{2}\\ 3x_{1}-x_{2}-\sin\;x_{2} \end{array}\right). \end{array}$$

This problem has a solution x* = (0,0)^*T*^. We test this problem by using initial value *x*_0_ = (0.3, −0.3)^*T*^ as a starting point. The test results are listed in Table 2.

**Example 3**

The test function is as follows (see [4]):

(67) $$\begin{array}{c} x_{1}^{2}+x_{2}^{2}+x_{3}^{2}-1=0,\\ 2x_{1}^{2}+x_{2}^{2}-4x_{3}=0,\\ 3x_{1}^{2}-4x_{2}^{2}+x_{3}^{2}=0. \end{array}$$

We test this problem by using *x*_0_ = (0.5,0.5,0.5)^*T*^. The test results are listed in Table 3.

**Example 4**

The test function is *F*(*x*) = (*f*_1_(*x*), *f*_2_(*x*), *f*_3_(*x*), *f*_4_(*x*))^*T*^, where *x* = (*x*_1_, *x*_2_, *x*_3_, *x*_4_)^*T*^ ∈ *R* ^4^ and *f*_*i*_ : *R* ^4^ → *R*, *i* = 1,2, 3,4, such that (see [9, 10, 13])

(68) f1(x)=x2x3+x4(x2+x3),f2(x)=x1x3+x4(x1+x3),f3(x)=x1x2+x4(x1+x2),f4(x)=x1x2+x1x3+x2x3−1.

This problem has two solutions: they are

(69) x∗=(−13,−13,−13,123)T,x∗∗=(13,13,13,−123)T.

We test this problem by using *x*_0_ = (−1, −1, −1, −1)^*T*^ (the iterative sequence converges to x*) and *x*_0_ = (1,1, 1, −2)^*T*^ (the iterative sequence converges to x**) as starting point, respectively. The test results are listed in Tables 4 and 5.

**Example 5**

The test function is as follows (see [10, 13, 14]):

(70) Fi(x)=xixi+1−1, i=1,…,n−1, Fn(x)=x1xn−1.

This problem has two solutions: they are x* = (1,1,…, 1)^*T*^ and x** = (−1, −1,…, −1)^*T*^. We test this problem by using *x*_0_ = (2,2,…, 2)^*T*^ (the iterative sequence converges to x*) and *x*_0_ = (−2, −2,…, −2)^*T*^ (the iterative sequence converges to x**) as starting point, respectively. The test results are listed in Tables 6 and 7, which are obtained for *n* = 101.

**Example 6**

Consider the unrestraint optimum problem (see [18])

(71) $$\begin{array}{c} \underset{x\in R^{n}}{\min}\;f\left(x\right), \end{array}$$

where *f* : *R* ^*n*^ → *R* is defined by

(72) $$\begin{array}{c} f\left(x\right)=\sum_{i=2}^{n}\left[{\left(x_{i-1}^{2}+x_{i}^{2}\right)}^{2}-4x_{i-1}+3\right]. \end{array}$$

By KKT condition we have

(73) $$\begin{array}{c} F\left(x\right)=\frac{1}{4}\nabla f\left(x\right)=0, \end{array}$$

where *F*(*x*) = (*F*_1_(*x*), *F*_2_(*x*),…, *F*_*n*_(*x*))^*T*^,

(74) F1(x)=x1(x12+x22)−1,Fi(x)=xi(xi−12+2xi2+xi+12)−1, i=2,…,n−1,Fn(x)=xn(xn−12+xn2).

We test this problem by using *x*_0_ = (1,1,…, 1)^*T*^ as starting point. The test results are listed in Table 8, which are obtained for *n* = 512.

**Example 7**

Consider the discrete two-point boundary value problems (see [19]):

(75) $$\begin{array}{c} F\left(x\right)=Ax+\frac{1}{{\left(n+1\right)}^{2}}G\left(x\right)=0, \end{array}$$

where *A* is an *n* × *n* tridiagonal matrix defined by

(76) $$\begin{array}{c} A=\left(\begin{array}{cccc} 2 & -1 & & \\ -1 & 2 & -1 & \\ & \ddots & \ddots & \ddots \\ & & -1 & 2 \end{array}\right), \end{array}$$

*G*(*x*) = (*G*_1_(*x*), *G*_2_(*x*),…, *G*_*n*_(*x*))^*T*^, and *G*_*i*_(*x*) = sin*x*_*i*_ − 1, *i* = 1,2,…, *n*. We test this problem by using *x*_0_ = (1,1,…, 1)^*T*^ as starting point. The test results are listed in Table 9, which are obtained for *n* = 1024.

## 5. Conclusion

From the seven examples in Section 4, we can see that the newly developed method (42)-(43) has the advantages of fast convergence speed (we can get from the CPU time), small number of iterations. Especially, the value of convergence order *p* that appears in Tables 2–7 is the highest compared to the other four methods. Although our method's convergence order is not always higher than the method of Cordero and Martínez and Torregrosa (NAd1), ours is superior in the number of iterations and CPU time to the other four methods. In a word, our method (42)-(43) is quite robust and effective.

## Figures and Tables

**Table 1:** Numerical results of Example 1 with initial value *x*_0_ = 4.

Method	Iterations	CPU time	Error	Convergence order (*p*)
NR1	6	0.0006	8.2276*e* − 009	2.95
NAd1	6	0.0007	7.1942*e* − 014	3.91
DV	7	0.0018	7.1783*e* − 012	2.98
CT	6	0.0005	7.1828*e* − 009	2.95
HM	6	0.0003	6.6613*e* − 015	3.00

**Table 2:** Numerical results of Example 2 with initial value *x*_0_ = (0.3, −0.3)^*T*^.

Method	Iterations	CPU time	Error	Convergence order (*p*)
NR1	3	0.0007	1.3502*e* − 006	3.15
NAd1	3	0.0011	1.4541*e* − 006	4.71
DV	4	0.0008	2.8501*e* − 013	3.02
CT	3	0.0007	1.3487*e* − 006	3.15
HM	3	0.0004	1.0359*e* − 014	5.69

**Table 3:** Numerical results of Example 3 with initial value *x*_0_ = (0.5,0.5,0.5)^*T*^.

Method	Iterations	CPU time	Error	Convergence order (*p*)
NR1	4	0.0008	5.5511*e* − 017	3.24
NAd1	4	0.0007	5.6038*e* − 006	4.51
DV	4	0.0007	0.0000*e* + 000	3.62
CT	4	0.0009	5.5511*e* − 017	3.24
HM	3	0.0005	3.8599*e* − 012	Inf

**Table 4:** Numerical results of Example 4 with initial value *x*_0_ = (−1, −1, −1, −1)^*T*^.

Method	Iterations	CPU time	Error	Convergence order (*p*)
NR1	4	0.0013	2.9032*e* − 014	3.32
NAd1	3	0.0011	5.6038*e* − 006	4.40
DV	4	0.0008	1.2469*e* − 011	3.38
CT	4	0.0015	2.9088*e* − 014	3.32
HM	3	0.0006	3.3483*e* − 008	4.68

**Table 5:** Numerical results of Example 4 with initial value *x*_0_ = (1,1, 1, −2)^*T*^.

Method	Iterations	CPU time	Error	Convergence order (*p*)
NR1	4	0.0016	2.9365*e* − 014	3.29
NAd1	3	0.0010	5.7635*e* − 006	4.13
DV	4	0.0008	1.2666*e* − 011	3.33
CT	4	0.0014	2.9421*e* − 014	3.29
HM	3	0.0006	3.4298*e* − 008	4.43

**Table 6:** Numerical results of Example 5 with initial value *x*_0_ = (2,2,…, 2)^*T*^.

Method	Iterations	CPU time	Error	Convergence order (*p*)
NR1	4	0.0104	2.6223*e* − 013	2.98
NAd1	3	0.0105	5.2789*e* − 006	3.23
DV	4	0.0072	6.9607*e* − 011	2.96
CT	4	0.0085	2.6223*e* − 013	2.98
HM	3	0.0047	4.6461*e* − 008	3.58

**Table 7:** Numerical results of Example 5 with initial value *x*_0_ = (−2, −2,…, −2)^*T*^.

Method	Iterations	CPU time	Error	Convergence order (*p*)
NR1	4	0.0093	2.6223*e* − 013	2.98
NAd1	3	0.0115	5.2789*e* − 006	3.23
DV	4	0.0078	6.9607*e* − 011	2.96
CT	4	0.0107	2.6223*e* − 013	2.98
HM1	3	0.0052	4.6461*e* − 008	3.58

**Table 8:** Numerical results of Example 6 for dimensions *n* = 512.

Method	Iterations	CPU time	Error	Convergence order (*p*)
NR1	4	0.1513	1.1567*e* − 013	3.24
NAd1	5	2.2769	1.1102*e* − 016	3.98
DV	5	0.1774	1.1102*e* − 016	2.90
CT	4	0.1823	1.1567*e* − 013	3.24
HM	4	0.1006	4.8272*e* − 013	3.77

**Table 9:** Numerical results of Example 7 for dimensions *n* = 1024.

Method	Iterations	CPU time	Error	Convergence order (*p*)
NR1	2	0.6050	3.0878*e* − 016	Inf
NAd1	2	2.5966	9.8532*e* − 016	Inf
DV	2	1.3221	9.2981*e* − 016	Inf
CT	2	0.7301	7.7716*e* − 016	Inf
HM	2	0.4460	2.4424*e* − 011	Inf

## References

[1] Potra FA, Pták V (1984). *Nondiscrete Induction and Iterative Processes*.

[2] Noor MA (2007). New iterative schemes for nonlinear equations. *Applied Mathematics and Computation*.

[3] Abbasbandy S (2003). Improving Newton-Raphson method for nonlinear equations by modified Adomian decomposition method. *Applied Mathematics and Computation*.

[4] Darvishi MT, Barati A (2007). A third-order Newton-type method to solve systems of nonlinear equations. *Applied Mathematics and Computation*.

[5] Chun C (2006). A new iterative method for solving nonlinear equations. *Applied Mathematics and Computation*.

[6] Chun C (2005). Iterative methods improving newton’s method by the decomposition method. *Computers and Mathematics with Applications*.

[7] Darvishi MT, Barati A (2007). Super cubic iterative methods to solve systems of nonlinear equations. *Applied Mathematics and Computation*.

[8] Golbabai A, Javidi M (2007). A new family of iterative methods for solving system of nonlinear algebric equations. *Applied Mathematics and Computation*.

[9] Cordero A, Torregrosa JR (2006). Variants of Newton’s method for functions of several variables. *Applied Mathematics and Computation*.

[10] Cordero A, Torregrosa JR (2007). Variants of Newton’s Method using fifth-order quadrature formulas. *Applied Mathematics and Computation*.

[11] Frontini M, Sormani E (2004). Third-order methods from quadrature formulae for solving systems of nonlinear equations. *Applied Mathematics and Computation*.

[12] Aslam Noor M, Waseem M (2009). Some iterative methods for solving a system of nonlinear equations. *Computers and Mathematics with Applications*.

[13] Cordero A, Martínez E, Torregrosa JR (2009). Iterative methods of order four and five for systems of nonlinear equations. *Journal of Computational and Applied Mathematics*.

[14] Cordero A, Hueso JL, Martínez E, Torregrosa JR (2010). Accelerated methods of order 2 p for systems of nonlinear equations. *Journal of Computational and Applied Mathematics*.

[15] Huang N, Ma CF, Liu ZG (2012). A new extragradient-like method for solving variational inequality problems. *Fixed Point Theory and Applications*.

[16] Burden RL, Faires JD (2001). *Numerical Analysis*.

[17] Ortega JM, Rheinboldt WC (1970). *Iterative Solution of Nonlinear Equations in Several Variables*.

[18] Yamakawa E, Fukushima M (1999). Testing parallel variable transformation. *Computational Optimization and Applications*.

[19] Moré JJ, Garbow BS, Hillstrome KE (1981). Testing unconstrained optimization software. *ACM Transactions on Mathematical Software*.

